# The Pocket-Creation Method Facilitates Endoscopic Submucosal Dissection of Gastric Neoplasms Along the Lesser Curvature at the Gastric Angle

**DOI:** 10.3389/fmed.2022.825325

**Published:** 2022-03-14

**Authors:** Masafumi Kitamura, Yoshimasa Miura, Satoshi Shinozaki, Alan Kawarai Lefor, Hironori Yamamoto

**Affiliations:** ^1^Department of Medicine, Division of Gastroenterology, Jichi Medical University, Shimotsuke, Japan; ^2^Shinozaki Medical Clinic, Utsunomiya, Japan; ^3^Department of Surgery, Jichi Medical University, Shimotsuke, Japan

**Keywords:** endoscopic submucosal dissection, pocket-creation method, stomach neoplasms, patient outcomes assessment, endoscopic mucosal resection

## Abstract

**Background:**

Endoscopic submucosal dissection (ESD) of superficial gastric lesions located along the lesser curvature at the gastric angle is a challenging situation due to paradoxical movement and a protruding angle. The pocket-creation method (PCM) can overcome this difficulty by stabilizing the tip of the endoscope in the pocket and minimizing insufflation of the stomach, which enables horizontal and straight dissection. This study aims to clarify whether the PCM improves the technical outcomes of ESD of superficial gastric neoplasms along the lesser curvature at the angle.

**Methods:**

From October 2006 to June 2021, 158 gastric lesions along the lesser curvature at the angle were resected with needle-type knives. We retrospectively reviewed the records and divided them into the PCM group (*n* = 61) and the conventional method (CM) group (*n* = 97). The primary outcome measurement was dissection speed (in mm^2^/min).

**Results:**

The two groups were not significantly different for baseline characteristics such as macroscopic type and size except for the proportion of adenomas. The proportion of expert endoscopists was not significantly different between the two groups (*P* = 0.141). The dissection speed was significantly faster in the PCM group than in the CM group (*P* = 0.001). There were no holes in the resected specimens in the PCM group, while five lesions in the CM group (5%) had a hole (*P* = 0.182). There were no significant differences in the incidence of adverse events.

**Conclusions:**

This is the first study to show that the PCM outperforms the CM for ESD of lesions located along the lesser curvature at the gastric angle. The PCM facilitated ESD of these lesions by significantly increasing dissection speed when a needle-type knife is used with no increase in adverse events.

## Introduction

Gastric endoscopic submucosal dissection (ESD) has been developed as a minimally invasive treatment for superficial gastric neoplasms with a high *en bloc* resection rate and preservation of postoperative gastric function. However, gastric ESD is still technically challenging for many endoscopists. Factors contributing to these difficulties include endoscopic maneuverability, the abundance of blood vessels, degree of submucosal fibrosis, fluctuations due to breathing and heartbeat, and so on. The stomach has a wide lumen and complex structure that make gastric ESD difficult. Greater difficulty when performing ESD results in lower quality of the resected specimens, longer procedure time, and a higher adverse event rate (1, 2).

**Figure 1:**
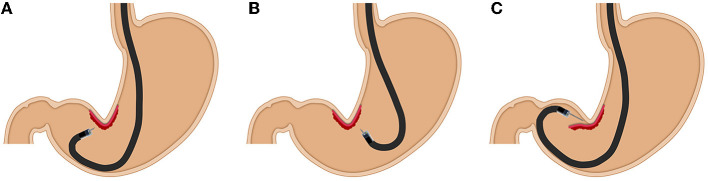
The difficulties of ESD using retroflexion. **(A,B)** A vertical approach is inevitable from the antrum and the lower body with retroflexion. **(C)** In an unreachable situation, endoscopists need to protrude the endoknife sheath into the gastric lumen.

In most gastric ESD procedures, retroflexion is generally used. Even using retroflexion, a distant and/or vertical approach to the muscularis is sometimes inevitable, especially on the lesser curvature at the gastric angle (Figure 1). Although the lesser curvature in the lower body is reported to be a location with a high rate of gastric cancers treated with ESD (3), ESD is technically difficult. Traction methods and multi-bending endoscopes are viable options to facilitate ESD under challenging circumstances (4–6). Zhang et al. reported the usefulness of endoscopic submucosal tunnel dissection for superficial gastric neoplasms along the lesser curvature larger than 3 cm with retroflexed approaches (7).

The pocket-creation method (PCM) is an innovative ESD strategy for achieving safe and high-quality ESD (8). The usefulness of PCM for ESD in the colon and duodenum have been reported (9–12). We have also reported the effectiveness of PCM for ESD of gastric lesions involving the pyloric ring (13). The PCM offers four distinct advantages: (1) the injected solution is not dispersed due to a minimal incision; (2) both traction and countertraction are obtained simultaneously because the transparent hood stretches the submucosal tissue in the pocket; (3) a vertical approach toward the muscularis can be changed to a tangential approach, and (4) the influence of cardiopulmonary movement is diminished due to synchronization of the endoscope and the pocket (14, 15).

This study aims to evaluate usefulness of the PCM for ESD of superficial gastric neoplasms located along the lesser curvature at the gastric angle compared to the conventional method (CM).

## Materials and Methods

### Study Population

**Figure 2:**
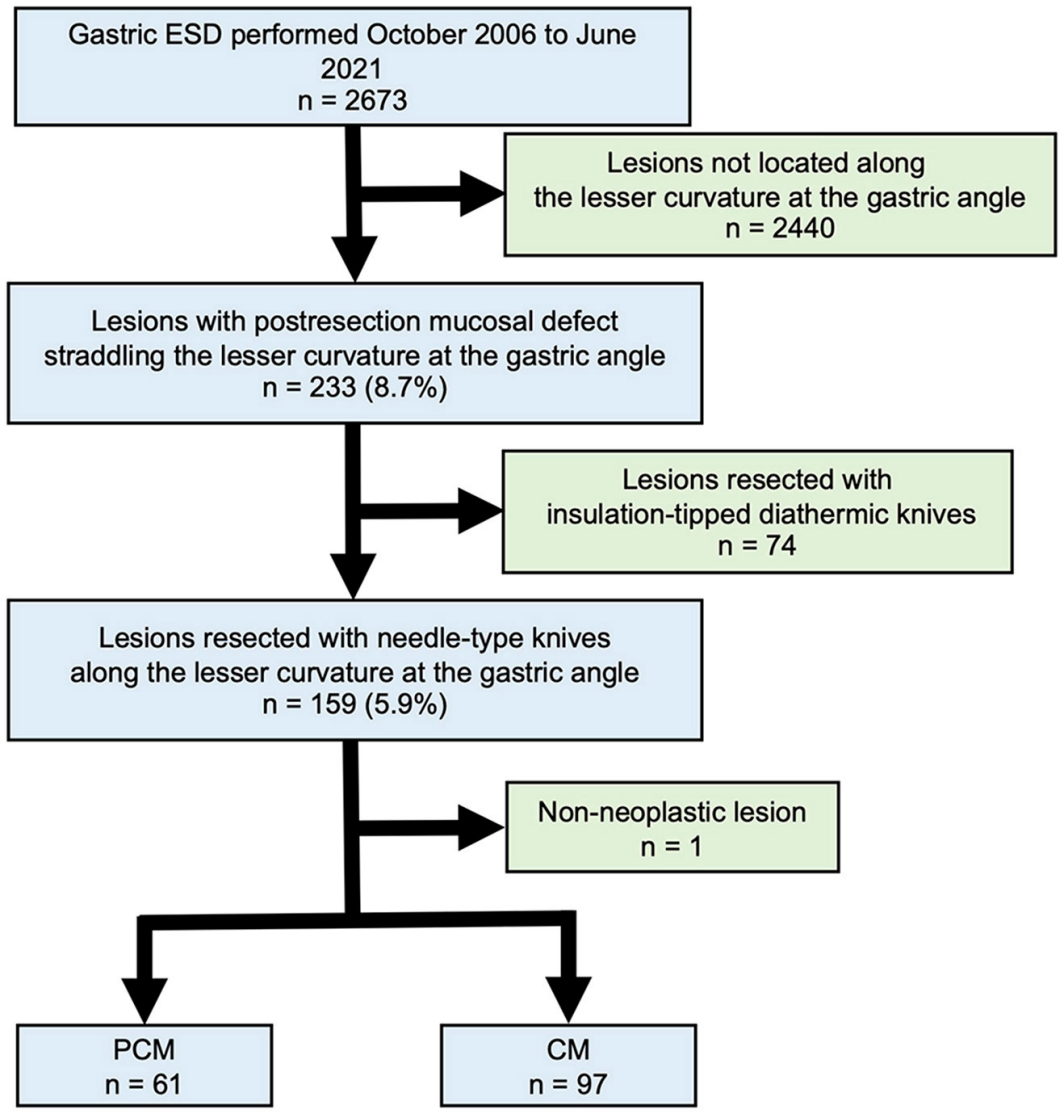
Study inclusion process.

From October 2006 to June 2021, 2673 gastric lesions treated using ESD at Jichi Medical University Hospital were reviewed and are included in this study. We abstracted the medical history, endoscopic findings, and pathologic findings from medical records that were evaluated retrospectively. The inclusion criterion was a gastric lesion with a postresection mucosal defect straddling the lesser curvature at the gastric angle. Exclusion criteria were non-neoplastic lesions and lesions resected with an insulation-tipped diathermic knife such as the SAFEKnife V (DK2518DV15, Fujifilm, Tokyo, Japan) and an IT knife (KD610L, Olympus, Tokyo, Japan). Of these 2673 lesions, 233 were located along the lesser curvature at the angle. Of 233 lesions resected, 74 resected with an insulation-tipped diathermic knife and 1 non-neoplastic lesion were excluded. The remaining 158 lesions in 158 patients were included in the final analysis (Figure 2). Informed consent regarding ESD was obtained from all patients. The primary outcome measurement was dissection speed (in mm^2^/min). Secondary outcome measures were the R0 resection rate and the occurrence of adverse events. This retrospective review was approved by Jichi Medical University's Institutional Review Board (No. 20-103).

### Method of Gastric ESD

Before undergoing ESD, all patients were hospitalized and given intravenous sedation with pethidine, diazepam, and/or midazolam. The border and dot markings were identified using a magnification endoscope (EG-590ZW or EG-600ZW; Fujifilm). ESD was performed using an endoscope with a waterjet instrument (EG-450RD or EG-580RD; Fujifilm), carbon dioxide insufflation, and a small-caliber-tip transparent (ST) hood (DH-15GR, DH-28GR, or DH-33GR; Fujifilm) or a cylindrical hood (D-201-10804; Olympus) connected to the endoscope tip. 0.4% sodium hyaluronate solution (MucoUp; Seikagaku, Tokyo, Japan) with 0.002%−0.004% indigo carmine and 0.002% epinephrine was employed for submucosal injection. A Flush knife BT (DK2618JB-15; Fujifilm), a DualKnife (KD-650Q; Olympus), or a needle-knife were used to make the mucosal incision and submucosal dissection. Hemostatic forceps (HOYA Corporation, Tokyo, Japan) were utilized to control bleeding. The electrosurgical generators used were an ICC 200 or a VIO300 D (ERBE Elektromedizin GmbH, Tübingen, Germany).

*En bloc* resection was defined as resection of an entire lesion as a single specimen. An *en bloc* resection with negative horizontal and vertical margins was classified as an R0 resection. The resected specimen's area was determined using the formula: major axis (mm)/2 × minor axis (mm)/2 × 3.14. The dissection time was measured from the beginning of cutting the mucosa to the end of the resection. The dissection speed was calculated with the following formula: the area of specimen (mm^2^)/dissection time (minutes).

Patients took nothing by mouth for the next few days after ESD except water. The next day, a second-look endoscopy was performed. Regular oral intake was initiated 2 days following ESD in the absence of symptoms or evidence of adverse events, as well as normal laboratory values, and the patient was discharged on the fourth post-procedure day. A perforation that occurred during the ESD technique was described as an immediate perforation. Delayed perforation was defined as a perforation that occurred after the ESD process was completed. Melena or hematochezia with a hemoglobin concentration reduction of more than 2 g/dL, needing transfusion for a hemoglobin concentration <7 g/dL, or requiring endoscopic hemostasis within 14 days following ESD were all considered delayed bleeding. More than 2 weeks following hospital discharge, all patients returned to the clinic for an explanation of the pathologic results.

### The Conventional Method and Pocket-Creation Method

**Figure 3:**
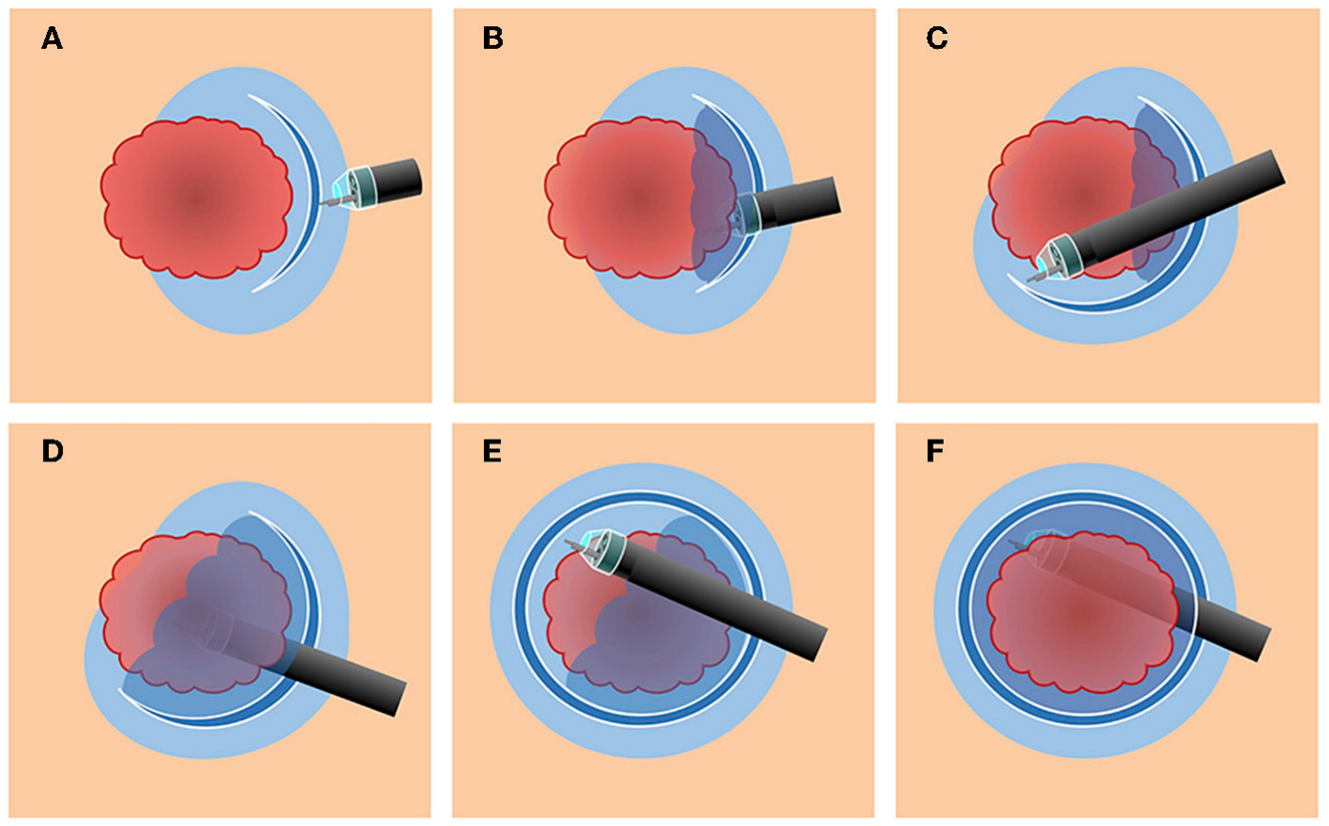
Schema of the conventional method. **(A)** A mucosal incision is made in the periphery at least 5 mm away from the lesion's margin. **(B)** Submucosal dissection is performed from the lateral edge of the tumor toward the center. **(C)** An additional mucosal incision is made in a step-by-step manner. **(D)** Submucosal dissection of the incised area is performed in the same manner. **(E)** A mucosal incision is made in the remaining area. **(F)** The remaining area is dissected, and an *en bloc* resection is accomplished.

In the CM, mucosal incision and submucosal dissection were repeated in a stepwise fashion (Figure 3). First, a mucosal incision is made in the periphery at least 5 mm away from the lesion's margin, followed by submucosal dissection from the lateral edge of the tumor toward the center. After dissecting all initial mucosal incision areas, additional mucosal incisions were made, and the same submucosal dissection was repeated. An antegrade approach or retroflexion were freely chosen depending on the ease of operation using these procedures.

**Figure 4:**
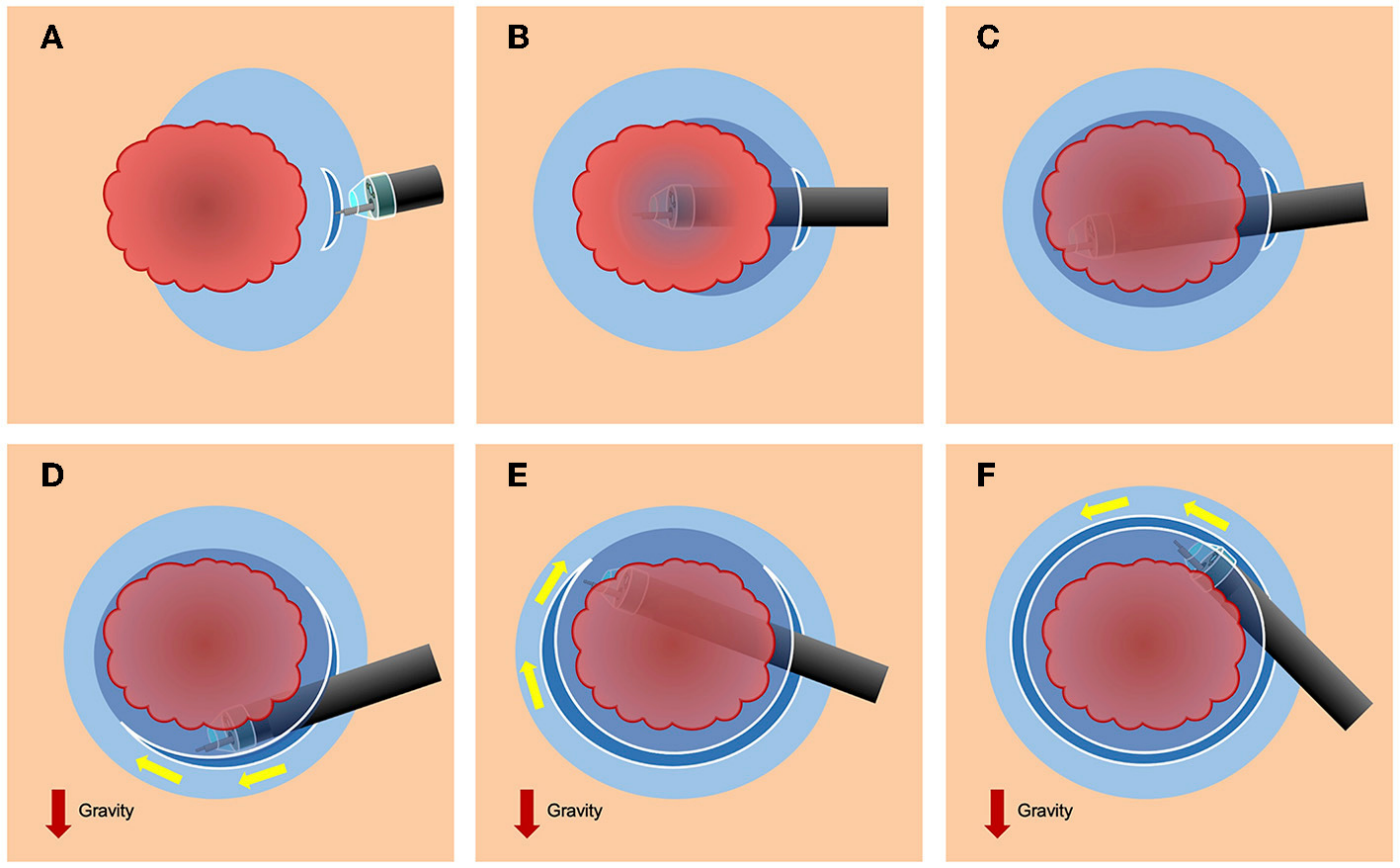
Schema of the pocket-creation method viewed from above. **(A)** A minimal mucosal incision is made at least 5 mm away from the lesion's margin. **(B,C)** A submucosal pocket is created under the tumor without a circumferential incision. **(D,E)** The pocket is opened in a step-by-step manner from the gravity side. **(F)** The remaining area is dissected, and an *en bloc* resection is accomplished.

**Figure 5:**
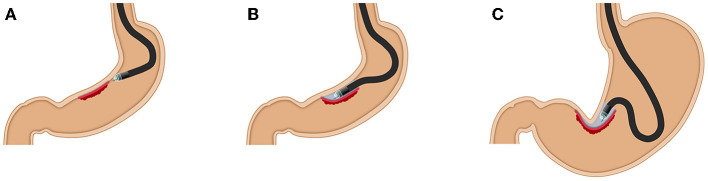
Schema of the pocket-creation method (PCM). **(A)** The PCM is started from proximal side of the tumor without retroflexion. **(B)** The PCM enables a tangential approach and straightforward dissection by aspirating gas and compressing the muscularis using strong traction and counter-traction in the pocket. **(C)** In the inflated stomach, a redundant loop of the endoscope results in a more acute gastric angle. Aspirating gas from the gastric lumen as much as possible is essential for a successful procedure.

**Figure 6:**
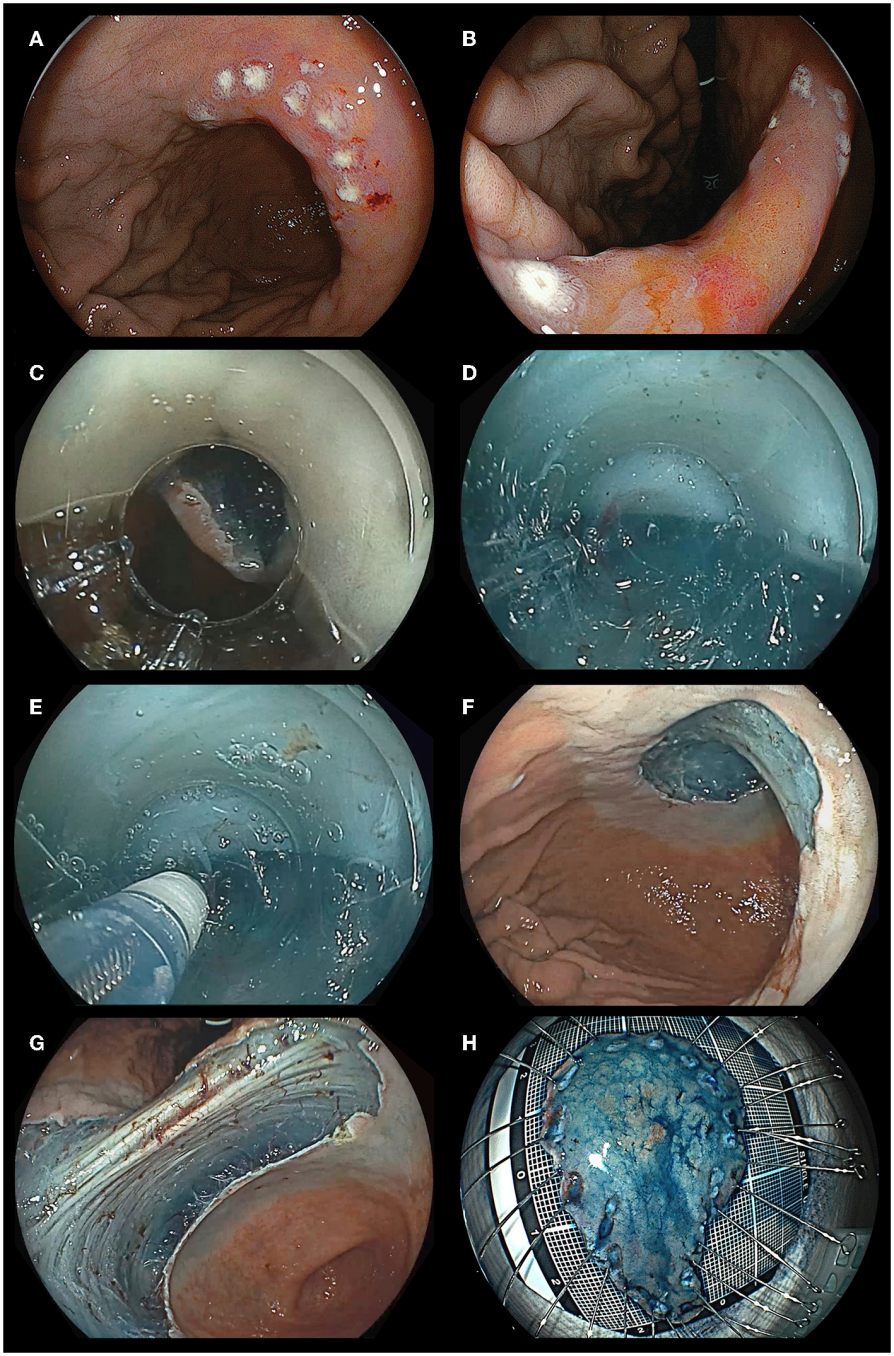
A representative case of endoscopic submucosal dissection with the pocket-creation method. **(A)** A depressed gastric lesion at the angle along the lesser curvature of the stomach. **(B)** Observation with retroflexion. **(C)** Creating a pocket entrance from the proximal side of the lesion with minimal insufflation. **(D)** Clear visualization of the submucosal layer in the pocket using a transparent hood enables selection of the dissection level by allowing the operator to recognize blood vessels and the muscularis. **(E)** Since the knife is led to the center of the endoscope's field of vision by the ST-hood, the dissection level of the submucosal layer can be adjusted even if the muscularis is at 12 o'clock in the endoscope field of vision. **(F)** The mucosal defect observed from looking down. **(G)** The mucosal defect observed with retroflexion. **(H)** The resected specimen is 61x38 mm in size. The pathology is a well-differentiated intramucosal cancer, with negative margins and without lympho-vascular invasion.

The PCM is started on the proximal side without retroflexion. First, a minimal mucosal incision is made at least 5 mm away from the lesion's margin (Figures 4, 5A,B, 6). Second, careful submucosal dissection is necessary to insert the endoscope tip into the pocket, and the pocket is subsequently enlarged without a circumferential incision to preserve traction and countertraction by the hood tip and prevent dispersion of the injected solution. Third, the pocket is opened entirely from the gravity side once the submucosal dissection under the tumor is completed (13). For a successful gastric ESD procedure with the PCM, it is critical to aspirate as much gas as possible from the gastric lumen (16). Aspirating gas from the gastric lumen can help the endoscope maneuver more efficiently and allow a tangential approach (Figures 5B,C). The PCM requires a small-caliber-tip transparent (ST) hood (DH-15GR, DH-28GR or DH-33GR; Fujifilm). The ST-hood is tapered with a narrow aperture that allows for easier entry into the submucosal area, and the hood's tip provides both traction and countertraction. Furthermore, the ST-hood contains a groove that guides the knife to the center of the endoscopic field of vision, which is critical for selecting the submucosal layer dissection level. The dissection level should be immediately above the muscularis to achieve a high-quality specimen with a thick submucosal layer.

The CM was carried out in the early phases of this study, from October 2006 to August 2014. The PCM was first performed in September 2014, and it has since been used virtually exclusively. An expert endoscopist was defined as was defined as performing more than 50 gastric ESD procedures.

### Statistical Analysis

All statistical analyses were carried out using EZR version 1.52 (Saitama Medical Center, Jichi Medical University, Saitama, Japan), which is a graphical user interface for R version 4.02 (The R Foundation for Statistical Computing Vienna, Austria). EZR is a modified version of R commander that adds statistical functions commonly used in biostatistics. The χ^2^ test was applied to assess the association between categorical variables, and the Mann–Whitney U test was used to compare mean values. A *P*-value < 0.05 was regarded as statistically significant.

## Results

### Baseline Characteristics of Patients and Gastric Lesions

The 158 lesions in the study were divided into two groups: PCM (*n* = 61) and CM (*n* = 97) (Table 1). The two groups were not significantly different for baseline characteristics such as macroscopic type and size except for the proportion of adenomas. The proportion of expert endoscopists who performed the procedures was not significantly different between the two groups (*P* = 0.141). Since the ST-hood is required for the PCM to provide both traction and counter-traction in the pocket, it was employed more frequently in the PCM group than in the CM group. Traction devices were not used in any procedures.

**Table 1:** Patient characteristics.

**Characteristic**	**Pocket-creation method**	**Conventional method**	***P* value**
Number of lesions	61	97	
Number of patients	61	97	
Age, median (range), years	73 (51–93)	73 (53–89)	0.693
Gender, male, *n* (%)	48 (79)	72 (74)	0.571
Macroscopic type			0.081
Elevated	27 (44)	58 (60)	
Flat or depressed	34 (56)	39 (40)	
Tumor diameter, median (range), mm	21 (5–84)	20 (4–70)	0.748
Pathologic findings, *n* (%)
Adenoma	0 (0)	12 (12)	0.011
Intramucosal carcinoma	54 (89)	78 (80)	0.263
Slightly invasive (<500 μm) submucosal carcinoma	1 (2)	2 (2)	1
Deeply invasive (≧500μm) submucosal carcinoma	5 (8)	5 (5)	0.668
Undifferentiated	1 (2)	0 (0)	0.814
Ulcer	12 (20)	19 (20)	1
Vascular invasion	7 (12)	6 (6)	0.379
Lymphatic invasion	5 (8)	4 (4)	0.470
Venous invasion	5 (8)	4 (4)	0.470
Hood			
Cylindrical hood	0 (0)	49 (50)	<0.001
DH-15GR	40 (66)	21 (22)	<0.001
DH-28GR	3 (5)	25 (26)	0.002
DH-33GR	18 (29)	2 (2)	<0.001
Performed by expert endoscopist	32 (53)	38 (39)	0.141
Antiplatelet agent use or anticoagulation	13 (21)	15 (16)	0.470

### Quality of ESD

The dissection speed was significantly faster in the PCM group than in the CM group (*P* = 0.001) (Table 2). Among expert endoscopists, the dissection speed was significantly faster in the PCM group than in the CM group (*P* = 0.003). The difference was not significant among non-expert endoscopists although the dissection speed tended to be faster in the PCM group (*P* = 0.256). There were no significant differences in R0 resection rates between the two groups. The PCM group had no holes in resected specimens, but the CM group had 5/97 lesions (5%) with holes (*P* = 0.182). There were no statistically significant variations in the rate of adverse events. Endoscopic hemostasis successfully controlled all delayed bleeding. Two cases of intra-procedural perforation occurred in the CM group, both successfully managed non-operatively by clip application. No perforations occurred in the PCM group.

**Table 2:** Clinical outcomes and adverse events.

**Characteristic**	**Pocket-creation method**	**Conventional method**	***P* value**
*En bloc* resection, *n* (%)	61 (100)	96 (99)	1
R0 resection, *n* (%)	55 (90)	88 (91)	1
Hole in the resected specimen, *n* (%)	0 (0)	5 (5)	0.182
Positive horizontal margin, *n* (%)	3 (5)	4 (4)	1
Positive vertical margin, *n* (%)	3 (5)	6 (6)	1
Positive horizontal and vertical margin, *n* (%)	5 (8)	9 (9)	1
Resected specimen diameter, median (range), mm	46 (16–110)	41 (18–91)	0.084
Dissection time, median (range), min	65 (10–256)	69 (6–232)	0.760
Dissection speed, median (range), mm^2^/min	20 (6–49)	14 (5–56)	0.001
Needs snare resection, *n* (%)	1 (2)	4 (4)	0.688
Delayed bleeding, *n* (%)	6 (10)	4 (4)	0.271
Perforation, *n* (%)	0 (0)	2 (2)	0.691

## Discussion

This study illustrates the usefulness of the PCM in ESD of superficial gastric neoplasms located along the lesser curvature at the gastric angle, which is a challenging procedure due to paradoxical endoscopic maneuverability and a protruding structure. When PCM was used, the dissection speed was significantly higher than when the CM was used. Considering our previous reports and the results of the present study, the PCM is a viable option to overcome difficulties in ESD of gastric lesions.

In an insufflated stomach, approaching a lesion along the lesser curvature at the angle may be unstable and unreachable, and a vertical approach is inevitable with retroflexion. In a distant approach to an unreachable situation, endoscopists need to protrude the endoknife sheath into the wide gastric lumen, making the movement rough and unstable. Dissecting the submucosa without identifying blood vessels and the muscularis increases the danger of bleeding and perforation in this situation. Even if the endoscopic approach is straightforward, a vertical approach is inevitable. The vertical approach makes dissection difficult and increases the risk of damaging the muscularis. The PCM can change a vertical approach to a tangential approach by entering the pocket in a way that facilitates stable submucosal dissection and hemostasis to minimize intraprocedural bleeding. Stabilization of the tip of the endoscope and clear visualization of the submucosal layer in the pocket enables the selection of the dissection level by recognizing blood vessels and the muscularis.

It is also noteworthy that there were no holes specimens resected using the PCM. When using the CM, an antegrade approach usually leads to a downhill approach at the gastric angle, and a retrograde approach with retroflexion causes a vertical approach against the lesser curvature at the angle. However, the PCM enables a tangential approach and straightforward dissection by compressing the muscularis using effective traction and counter-traction in the pocket. Minimum insufflation of the stomach during the PCM makes the gastric angle less acute which facilitates an antegrade and straightforward submucosal dissection.

We acknowledge that this study has limitations. First, this is a retrospective, single-center observational study. A prospective, randomized controlled trial may be desirable to verify these results. Second, there was a time frameshift for the two groups. The evolution of endoscopes and devices and the learning curve effect may have influenced the results. Third, this study did not compare ESD using insulated tip knives or traction devices. International multicenter collaboration is needed for the standardization and development of gastric ESD.

The PCM is a simple technique to accomplish sophisticated ESD and is more effective for endoscopists familiar with ESD procedures. The PCM may facilitate gastric ESDs in various locations such as the fornix, along the lesser curvature, and the pyloric ring (13, 16).

In conclusion, the PCM facilitated gastric ESD along the lesser curvature at the gastric angle by increasing dissection speed when a needle-type knife is used. The results of this retrospective study justify future prospective multicenter studies using the PCM in gastric ESD, which vary greatly in difficulty depending on the location of lesion.

## Data Availability Statement

The original contributions presented in the study are included in the article/supplementary material, further inquiries can be directed to the corresponding author.

## Ethics Statement

The studies involving human participants were reviewed and approved by Jichi Medical University. The patients/participants provided their written informed consent to participate in this study.

## Author Contributions

MK, YM, SS, and HY contributed to the conception and design of the study. MK and YM organized the database and performed the statistical analysis. MK wrote the first draft of the manuscript. YM, SS, AL, and HY wrote sections of the manuscript. All authors contributed to manuscript revision, read, and approved the submitted version.

## Conflict of Interest

HY has a consultant relationship with Fujifilm Corporation and has received honoraria, grants, and royalties from the company. YM has received honoraria from Fujifilm Corporation. This study received no funding from Fujifilm Corporation nor any other funding source. The remaining authors declare that the research was conducted in the absence of any commercial or financial relationships that could be construed as a potential conflict of interest.

## Publisher's Note

All claims expressed in this article are solely those of the authors and do not necessarily represent those of their affiliated organizations, or those of the publisher, the editors and the reviewers. Any product that may be evaluated in this article, or claim that may be made by its manufacturer, is not guaranteed or endorsed by the publisher.
